# Registration and Nursing Reforms

**Published:** 1920-01-24

**Authors:** 


### THE MATRONS' AND SISTERS' DEPARTMENT.

### REGISTRATION AND NURSING REFORMS.

From the point of view of the Ministry of Health, which may be said to be the point of view of the public generally, the kernel of nursing reform lies in the interests of the patients. An ideal system of nursing would ensure an evenly distributed supply of nurses in institutions, nurses in districts, nurses in factories and schools, nurses in private families, all paid on an economic basis so that they were able to secure an independent old age, all working no more than the number of hours per day or week which experience has discovered to be generally adapted to their powers, all living under conditions which tend to conserve their energy and render them useful auxiliaries in the health services of the country. It is bracing sometimes to drop out of consideration the personal aspects of nursing reform, those which touch the individuals, and concentrate solely on the position of nurses as an asset to the nation. If nursing can be raised to its highest powers in the interests of the public, all private grievances will automatically disappear. Underpayment, overwork, and bad surroundings react immediately and even more disastrously on the patients than on the nurses.

The writer was once informed at a hospital not particularly noted for consideration towards its probationers that the midwifery instructors complained no nurse was of any use to them or to the patient after twelve or fourteen hours continuous work. This mild statement sheds a flood of light over the conditions which govern the pupil midwife's initiation into her duties. But which, after all, suffers most, the midwifery pupil who is kept out of her bed nominally learning her craft until she becomes dazed and unreliable, or the mothers who are attended in their great moment by women whose faculties are half suspended? Presumably some necessary experience is to be gained at each delivery the pupil attends, since the regulations enforce a minimum number. How much does a pupil learn or remember in the feverish torpor which seizes the mind after it has been strained beyond its powers of concentration? The pupil may sleep off her fatigues and scrape through her examinations. But she will not be as good a midwife as she might have been if trained under less foolish conditions. It is the mothers who bear the onus of a bad system.

We have instanced these notorious abuses in midwifery schools as the most striking examples of overwork reacting on the patients. But visit any Poor-law infirmary or understaffed hospital in the early hours of the morning, when probationers and superintendent have been on their feet for twelve hours continuously without any satisfactory break, and judge whether the patients are not suffering under the wretchedness of their tired nurses. We would go farther. Visit the room of a private patient rejoicing in the entire possession of two nurses, a patient whose grave conditions demand that he shall not be left for a moment, and judge whether the woman who has spent twelve continuous hours in the atmosphere of the sick-room, for weeks at a time, is physically and mentally at her best. The patient may be paying something like twelve guineas a week for his nurses. He is less well-nursed than he would be in an institution where, under an eight-hour day, the nurses were always fresh and alert.

The value of registration is that it brings the nursing services of the country a step forward in the business of standardisation. Year after year it is our privilege to chronicle great advances in the leading hospitals, as last week in the Royal Southern at Liverpool. The leading Poor-law infirmaries sometimes follow suit and sometimes, quite often in fact of late, they set an example. And yet, and yet, the great majority of institutions employing nurses remain as impervious to reform as the state of the labour market will permit. Every abuse which has for years been exposed in our columns still flourishes. Nurses are still ill-housed, ill-fed, ill-paid, overworked. In despair the nursing reformer casts aside the ideal of a healthy variety of administration and sighs for such a system of standardisation as shall at any rate obliterate authoritatively the worst evils under which the training-schools groan.

What could registration do to advance the millennium? It has a strong weapon in the acceptance or rejection of institutions as training-schools for nurses. The Board can refuse to sanction training which has been carried out under conditions which wear down the energies of the nurses and is likely to hinder them from the adequate discharge of their duties towards the patients. It can adopt a minimum standard of working hours having due regard to all the circumstances. It may later on expect managers to adhere to a scale of reasonable remuneration in the institutions on which it confers a cachet. And it may set up such a standard of wholesome living among nurses as is necessary in the interests of the patients to be observed.

